# Heading Date Is Not Flowering Time in Spring Barley

**DOI:** 10.3389/fpls.2017.00896

**Published:** 2017-05-30

**Authors:** Ahmad M. Alqudah, Thorsten Schnurbusch

**Affiliations:** HEISENBERG-Research Group Plant Architecture, Leibniz Institute of Plant Genetics and Crop Plant Research (LG)Seeland, Germany

**Keywords:** heading date, barley, stage-specific, genetic, QTL

## Introduction

**Figure 1:**
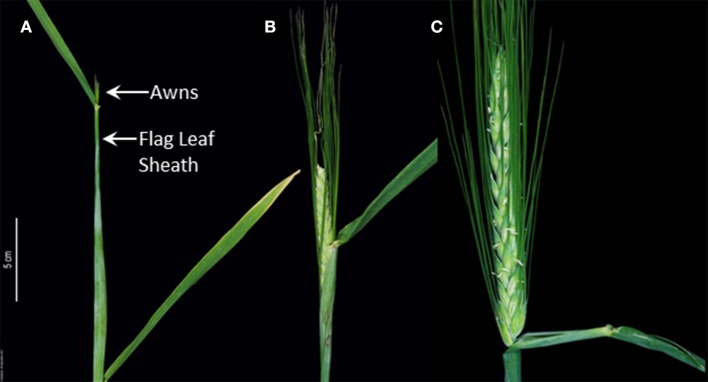
Different barley developmental stages according to the Zadoks scale. **(A)** At around Awn Tipping (i.e., –Z49) most barley plants (spring barley) pass through anthesis, while the spike is still enclosed in the flag leaf sheath. This is the actual “flowering time” stage of spring barley because fertilization happens now. **(B)** Spike heading (Z55; half of spike emerged) and, **(C)** anther extrusion (Z65) occur later and usually do not coincide with the fertilization events.

Understanding the stages of floral development is one of the major goals of crop breeding to produce new varieties that are better adapted to environmental cues with improved yield. The phase transition is a quantitative trait that is predominantly genetically controlled with a complex genetic network integrating endogenous and environmental factors. In recent years, it has become evident that the heading date in small grain cereal crops is one important stage that has been extensively studied and highly associated with environmental adaptation and yield. Heading date has a complex genetic architecture that makes it a target trait in barley (*Hordeum vulgare* L.) breeding programs. Fine adjustment of heading date is important for understanding other developmental traits such as leaf area, plant height, tillering, and grain number (Li et al., 2006; Alqudah et al., 2016). In addition, it is also considered as a decisive stage for improving yield and yield components (Esparza Martínez and Foster, 1998; Li et al., 2006; Cuesta-Marcos et al., 2009; Pasam et al., 2012). The timing of heading in barley has a substantial impact on range-wide eco-geographical adaptation and improving the yield, which is clearly shown in accessions from North-Western Europe and North America (with reduced response to long days), that are late in heading (Turner et al., 2005). This adaptation habit allows the barley plants to extend their vegetative phase that in turn increases biomass accumulation and grain yields (Turner et al., 2005). However, there appears to be confusion among barley researchers when the barley inflorescence (i.e., spike) shows the heading [Zadoks, Z50–Z59 (spike out of the flag leaf sheath), Figure 1B] and awn tipping appearance [(Z49), Figure 1A]. Such confusion can ultimately lead to inaccurate phenotypic results and interpretation especially in the context of identified stage-specific QTL/transcriptomes, or underlying genes.

## Phase transition in barley

The transition of major phases: vegetative, reproductive, and finally grain filling phase have been extensively studied for a wide range of aspects based on the external morphological appearance of immature or mature barley spikes (Zadoks et al., 1974; Kirby and Appleyard, 1987). Following germination, the vegetative phase (leaf and tiller formation) starts and proceeds until the collar is formed (Kirby and Appleyard, 1987; Sreenivasulu and Schnurbusch, 2012). During this phase, barley plants rapidly increase their biomass growth, while an extended vegetative phase results in a decrease in grain yield and survival of spikelets (Kitchen and Rasmusson, 1983). Subsequently, the switch from vegetative shoot apical meristem to inflorescence meristem identity is the sign of the transition to the reproductive phase which consists of two sub-phases. The early reproductive phase (spikelet initiation) that starts from double ridge to awn primordium stage; and the late-reproductive phase (spike growth and development) that starts from awn primordium to grain-filling via awn tipping and heading stages (Kirby and Appleyard, 1987; Sreenivasulu and Schnurbusch, 2012; Alqudah and Schnurbusch, 2014; Alqudah et al., 2014). The visibility of first awn primordia can be considered as the transition point from early- to late-reproductive phase; whereas anthesis or fertilization in spring barley usually happens at around the awn tipping stage (i.e., ~Z49). In winter barley, anthesis or fertilization mostly occurs after Z49. The late-reproductive phase, which includes the period from awn primordium to awn tipping Z49, is the longest developmental sub-phase in terms of the period that has a decisive impact on architectural traits, such as spikelet survival and final grain yield (Alqudah and Schnurbusch, 2014; Alqudah et al., 2014). Ultimately, the grain-filling phase starts soon after anthesis or fertilization with the onset of dry-matter accumulation for the developing grains and ends at the maturity stage. Hence, each of the developmental phase has a particular role in barley growth, development, and yield.

## What is the main sign of heading stage in barley?

An important question in heading date research is which developmental sign can be visualized for scoring it? And is there a chance to be confused with other developmental stages? According to the Zadoks scale (Zadoks et al., 1974); the most extensively used (more than 4,400 citations, Web of Science™) and accurate scale in temperate cereals (wheat and barley); heading stage is the appearance/emergence of the spike out of the flag leaf sheath [i.e., Z50–Z59 = first spikelet of inflorescence just visible to emergence of complete inflorescence, Figure 1B (half of spike emerged)]. Heading stage is commonly confused with the first awns appearance stage [awn tipping; Z49 which belongs to the booting phase (Z40–49), Figure 1A]. Note: Awn tipping can only be applied in awned cultivars; whereas in the awnless cultivars heading can only be scored at heading (Z50–Z59). Awn tipping (Z49) is the actual “flowering time” stage of spring barley because anthesis/fertilization happens around this stage. Most importantly, heading stage (i.e., Z50–59) does not mean “flowering time” in spring barley; likewise, it is not anther extrusion (i.e., Z60–69, Figure 1C; Zadoks et al., 1974) that usually occurs after heading and does not coincide with the fertilization events. Both previous stages are much later than the actual “flowering time,” i.e., anthesis/fertilization at ~Z49. Therefore, description on the sign and usage of the term “heading time” becomes an important point to be clarified for further studies. In the past there is a mix between these developmental stages; for instance, awn appearance (Z49) was used as a proxy for heading date (Igartua et al., 1999; Cuesta-Marcos et al., 2007; Casao et al., 2011; Digel et al., 2015; Maurer et al., 2015). Alqudah et al. (2014), however, demonstrated that these stages are morphologically/developmentally different and are partially under different genetic control. This finding strongly indicated that it is not plausible to use flowering/anther extrusion or awn tipping stages to assign the heading stage in spring barley.

Notably, the Zadoks scale fits very well in the case of wheat, where anthesis/fertilization occurs after the heading phase Z50–59 and coincides with the onset of anther extrusion, i.e., Z60–69 (Zadoks et al., 1974).

## How can scientists quantify the heading date?

The heading date is influenced by environmental conditions, and it is generally presented as required number of days to reach that stage. Environmental cues, however, do have a significant influence on growth and development, particularly temperature, where thermal time (growing degree-days GDD [°C^*^d]) is the most accurate and widely accepted way to present the actual required time to reach that stage (Slafer et al., 2003; Borras-Gelonch et al., 2010; Alqudah et al., 2014). GDD is an approach to calculate the temperature sums over time that can be used to assign and describe developmental and biological processes in crops such as wheat, barley, and maize (McMaster and Wilhelm, 1997; Miller et al., 2001). The importance of GDD lies in collecting daily average temperature (accumulated required temperature over a period of time to reach heading date) without looking at the total number of days to reach the stage (McMaster and Wilhelm, 1997; Miller et al., 2001). GDD is more reliable, steady, and robust for particular developmental stages across a season/environment (Slafer et al., 2003) and such an approach is crucial for further molecular and genetic analyses.

## Genetic studies of heading date in barley

In the last decade, extensive progress has been made in order to understand the genetic and molecular regulation of heading date in barley. *PHOTOPERIOD RESPONSE LOCUS 1* (*Ppd-H1*) is one of the central regulators for heading date in barley as a long-day (LD) crop. *Ppd-H1* encodes a component of the photoperiod pathway and the dominant alleles promote time to heading (Turner et al., 2005). The FLOWERING LOCUS T 1 (HvFT1) protein acts as the main integrator of the photoperiod and vernalization pathways; it has been considered as the main barley *FT*-like gene involved in the transition from the vegetative-to-reproductive phase under LD conditions (Faure et al., 2007). The transition from the vegetative to the reproductive phase is promoted by *Vrn-H1* in barley (Hemming et al., 2008); whereas *Vrn-H2* (*HvZCCT*) acts as suppressor heading in barley plants that have not been exposed to vernalization (Karsai et al., 2005; Casao et al., 2011). Moreover, Alqudah et al. (2014) found association signals for barley heading time with many of the genes encoding CCT (CONSTANS, CONSTANS-LIKE, and TIMING OF CAB1)-domain proteins. The evolutionarily conserved module of the photoperiod pathway under LD conditions, *GIGANTEA* (*GI*)-*CO*-*FT*, is also active in barley (Cockram et al., 2007). Independent of environmental cues, *EARLINESS PER SE* (*EPS*) genes control heading time and phase transition; but are poorly understood until now. Even though, dozens of heading date QTLs distributed over seven barley chromosomes have been detected using segregating populations (Esparza Martínez and Foster, 1998; Karsai et al., 2005; Li et al., 2006; Szucs et al., 2006; Cuesta-Marcos et al., 2009), or genome-wide association studies (Pasam et al., 2012; Alqudah et al., 2014; Maurer et al., 2015), besides the aforementioned major vernalization and photoperiod genes, only a few have been characterized in barley such as *EARLY FLOWERING 3* (*HvELF3*/*eps1*; Faure et al., 2012; Zakhrabekova et al., 2012; Boden et al., 2014), barley *CENTRORADIALIS* (*HvCEN*/*eps2*; Comadran et al., 2012), barley *LUX ARRHYTHMO*/*PHYTOCLOCK 1* (*HvLUX*/*PCL1*/*eps3*; Campoli et al., 2013; Gawronski et al., 2014), or barley *PHYTOCHROME C* (*HvPHY-C*/*eps5*; Nishida et al., 2013; Pankin et al., 2014).

Since several sophisticated next-generation sequencing or genetics approaches are now available to better analyze and understand the underlying molecular mechanisms of developmental traits, defining the correct developmental stage is very critical for understanding the molecular-genetic basis and biological pathways.

## Does the inaccuracy of scoring heading date affect QTL detection?

The previous genetic studies found that pre-heading phases are partially independent and are under different genetic control compared to stage/phase-specific QTLs or genetic mechanisms regulating heading stage in barley (Borras-Gelonch et al., 2010) and rice (Zhou et al., 2001). Moreover, researchers confirmed these conclusions and detected several stage-specific QTLs for awn tipping and heading stages, while some of these QTLs were shared between pre-heading and heading stages (Alqudah et al., 2014; Maurer et al., 2015). Alqudah et al. (2014) also showed that not all of the known associated heading date genes at awn tipping can be associated with heading date and vice versa. These findings strongly indicate that the accuracy of identifying heading date is crucial for detecting accurate stage-specific genetic factors underlying heading time variation.

## Concluding remarks

The investigations conducted so far on heading date clearly show that it is a key developmental stage for improving yield and adaptation. This article is intended to emphasize the importance of an accurate heading date definition in spring barley that in turn determines stage-specific genetic factors. Therefore, a clear morphological definition of heading date is key for further molecular and genetic analyses such as QTL/gene detection in addition to transcriptome and expression analyses. We put valuable inputs forward that will greatly help the scientific community working in this field.

## Author contributions

The authors confirmed their contribution of this work and prepared it for publication. AA formulated the idea. AA and TS wrote the manuscript.

### Conflict of interest statement

The authors declare that the research was conducted in the absence of any commercial or financial relationships that could be construed as a potential conflict of interest. The reviewer PMAK and handling Editor declared their shared affiliation, and the handling Editor states that the process met the standards of a fair and objective review.
